# Investigating calcification-related candidates in a non-symbiotic scleractinian coral, *Tubastraea* spp.

**DOI:** 10.1038/s41598-022-17022-4

**Published:** 2022-08-06

**Authors:** Laura Capasso, Manuel Aranda, Guoxin Cui, Melanie Pousse, Sylvie Tambutté, Didier Zoccola

**Affiliations:** 1 Marine Biology Department, Centre Scientifique de Monaco (CSM), 8 Quai Antoine 1er, Monte Carlo, 9800 Monaco; 2 Sorbonne Université, Collège Doctoral, 75005 Paris, France; 3 Marine Science Program, Biological and Environmental Sciences and Engineering Division, King Abdullah University of Science and Technology (KAUST), Thuwal, 23955-6900 Kingdom of Saudi Arabia; 4 Red Sea Research Center Center, King Abdullah University of Science and Technology, Thuwal, 23955-6900 Kingdom of Saudi Arabia; 5 Université Côte d’Azur, CNRS, Inserm, Institut for Research On Cancer and Aging, Nice (IRCAN), Medical School of Nice, Nice, France

**Keywords:** Physiology, Transcriptomics, Marine biology

## Abstract

In hermatypic scleractinian corals, photosynthetic fixation of CO_2_ and the production of CaCO_3_ are intimately linked due to their symbiotic relationship with dinoflagellates of the Symbiodiniaceae family. This makes it difficult to study ion transport mechanisms involved in the different pathways. In contrast, most ahermatypic scleractinian corals do not share this symbiotic relationship and thus offer an advantage when studying the ion transport mechanisms involved in the calcification process. Despite this advantage, non-symbiotic scleractinian corals have been systematically neglected in calcification studies, resulting in a lack of data especially at the molecular level. Here, we combined a tissue micro-dissection technique and RNA-sequencing to identify calcification-related ion transporters, and other candidates, in the ahermatypic non-symbiotic scleractinian coral *Tubastraea *spp. Our results show that *Tubastraea *spp. possesses several calcification-related candidates previously identified in symbiotic scleractinian corals (such as SLC4-γ, AMT-1like, CARP, etc.). Furthermore, we identify and describe a role in scleractinian calcification for several ion transporter candidates (such as SLC13, -16, -23, etc.) identified for the first time in this study. Taken together, our results provide not only insights about the molecular mechanisms underlying non-symbiotic scleractinian calcification, but also valuable tools for the development of biotechnological solutions to better control the extreme invasiveness of corals belonging to this particular genus.

## Introduction

In scleractinian corals (Cnidaria, Anthozoa), also known as stony corals, calcification leads to the formation of a biomineral composed of two fractions, one made of calcium carbonate (CaCO_3_) in the mineral form of aragonite^1–3^, and the other made of organic molecules^4–6^. Based on the ability of scleractinian corals to build reef structures, they are functionally divided into two main groups, namely, hermatypic (i.e. reef-building) and ahermatypic (i.e. non-reef-building). The majority of hermatypic corals hosts symbiotic dinoflagellates of the Symbiodinacae family^7^ in their tissues, commonly known as zooxanthellae^8^. This symbiotic association, which is lacking in most ahermatypic corals, provides the nutritional foundation for the host metabolism and boosts calcification in nutrient-poor tropical waters^9^.

Given the ability of hermatypic corals to build reefs, and given the economic and ecological importance associated with reef structures^10^, symbiotic scleractinian corals have been a major focus of calcification research over the years^2,11^. Whereas, ahermatypic non-symbiotic scleractinian corals have not been extensively studied and to date they remain under-represented especially in terms of molecular data^12^. These corals, however, should not be neglected as they represent important resources for scleractinian calcification research. This is because, in symbiotic scleractinian corals, calcification is linked to the photosynthetic fixation of CO_2_—both at the spatial as well as the temporal scales—which makes it difficult to disentangle these processes. Whereas, non-symbiotic scleractinian corals allow studying the transport mechanisms involved in calcification without the confounding factor of symbiosis^13^. In addition, studying calcification in non-symbiotic scleractinian corals further allows obtaining comparative information on the different scleractinian calcification strategies, therefore aiding in a better understanding of how calcification evolved within this order.

One of the main questions surrounding scleractinian calcification is how (i.e. via which molecular tools) corals promote a favorable environment for calcification^14^. As in other biological groups, coral calcification is a biologically controlled process, meaning that the precipitated mineral is not a byproduct of metabolic processes (also known as biologically induced biomineralization), but rather under strict biological and physiological control^15,16^. This control is exerted by a specialized tissue called the calicoblastic epithelium, that comprises the calcifying calicoblastic cells^17^. These cells control and promote calcification by modifying the chemical composition at the sites of calcification, which comprise intracellular vesicles and the extracellular calcifying medium (ECM)^14^. As recently suggested, calcification begins with the formation of amorphous calcium carbonate (ACC) nanoparticles, within intracellular vesicles, in the calicoblastic cells. ACC nanoparticles are then released via exocytosis into the ECM^18^. Here, ACC nanoparticles attach (i.e. nanoparticle attachment) and crystallize, while ions fill the interstitial spaces between them (i.e. ion-by-ion filling). Both, nanoparticle attachment and ion-by-ion filling processes require the calicoblastic cells to regulate ion transport and their concentration at the sites of calcification^2,19^. Furthermore, the calicoblastic cells also secrete an organic matrix which may stabilize ACC in the intracellular vesicles and play other roles, such as aiding and promoting ACC crystallization^20–24^.

Ion (i.e. calcium, carbonate, protons, and others) transport, to and from the sites of calcification, is of particular interest^2,19^. For instance, calcium and carbonate ions, the building blocks of the coral skeleton, have to be constantly supplied to the sites of calcification to sustain its growth^25^. Whereas, protons must be removed from the sites of calcification to increase the aragonite saturation state, prevent dissolution of calcium carbonate nanoparticles, and promote ion-by-ion filling mechanisms^14^.

Over the years, the ion transport model underlying scleractinian calcification has been well characterized for the calicoblastic cells through physiological and molecular studies^26–28^. However, such understanding is only partial and many calcification-related ion transporters still need to be identified. When searching for calcification-related candidates, different approaches are possible. One is the so called “targeted” approach and is based on the analysis of genes and/or proteins that have been chosen a priori- generally based on known biological functions in other model systems. This approach is extremely powerful for studying the genetic architecture of complex traits, such as calcification, in addition to being an effective approach for direct gene discovery^29^. Nevertheless, although the targeted approach has led to the identification of some of the most relevant calcification-related candidates in scleractinian calcification^13,30–32^, it is largely limited by the requirement of existing knowledge about the gene(s) under investigation. To overcome this limitation, other approaches, the so-called “broad” approaches, have been developed throughout the years. Broad approaches have the potential to discover novel candidates and pathways that have not been previously considered in the context of calcification, thus allowing a more holistic understanding of the process. These approaches have been performed at different levels, including the transcriptomic one, which relies on the use of RNA-sequencing (RNA-seq) technology^33–35^. To date, however, the use of RNA-seq to identify calcification-related candidates has been limited to analyzing coral molecular responses to environmental parameters known to influence calcification (such as light^33^ and CO_2_^36^), and only one study, performed in the symbiotic scleractinian coral *Stylophora pistillata*, has analyzed genes being more highly expressed in the coral calcifying tissue^37^.

Therefore, given the high potential of broad approaches in discovering novel candidates, and given the scarce amount of data available for non-symbiotic scleractinian corals^12^, we have performed, in this study, RNA-seq on coral species belonging to the ahermatypic non-symbiotic scleractinian genus *Tubastraea* (Lesson, 1829)^38^. *Tubastraea* corals include invasive saltwater species^39–42^ that were introduced into the southwestern Atlantic on oil platforms^42^. Since the late 1980s, these corals have been colonizing the rocky shores of the southeastern Brazilian coast^40^. Their rapid spread and growth provides them a competitive advantage and, therefore, represent a serious risk for endemic biodiversity loss^43^. In the absence of innovation in control methods, the dispersal of *Tubastraea* is expected to continue. In this context, calcification studies are fundamental to a better understanding of the life histories and population ecology of this genus. Of particular interest is the rapid linear skeletal growth of *Tubastraea* that could increase the competitiveness of these species^44^. In this study, we searched for calcification-related candidates, by sequencing the whole transcriptome from total colonies and oral fractions (i.e. fractions devoid of the aboral tissues that contain the calicoblastic cells) of *Tubastraea *spp.*,* obtained through a tissue micro-dissection technique. After assembling and annotating a highly complete transcriptome for *Tubastraea *spp., we identified and analyzed genes enriched in the total colony transcriptomes compared to the oral fraction transcriptomes. The analysis included both a comparison with calcification-related candidates previously characterized in symbiotic scleractinian corals, as well as a search for novel calcification-related ion transporter candidates.

This study provides insights into the molecular mechanisms underlying non-symbiotic scleractinian calcification and identifies valuable tools for the development of biotechnological solutions to better control the extreme invasiveness of corals belonging to this genus.

## Results

### Sequence read data and raw data pre-processing

RNA sequencing was performed for two sample groups, total colony and oral fraction (i.e. fraction devoid of the aboral tissues containing the calcifying calicoblastic cells), of three independent biological replicates (*n* = 3) of *Tubastraea *spp. Both groups, obtained through a previously developed micro-dissection protocol^45^, produced a total of 539,331,300 raw reads with an average of 44.9 ± 8.7 (mean ± SD) million read pairs per sample. Raw reads were subjected to quality trimming, which included adaptor removal, yielding a total of 369,357,576 trimmed reads.

### De novo transcriptome assembly and quality assessment

**Figure 1:**
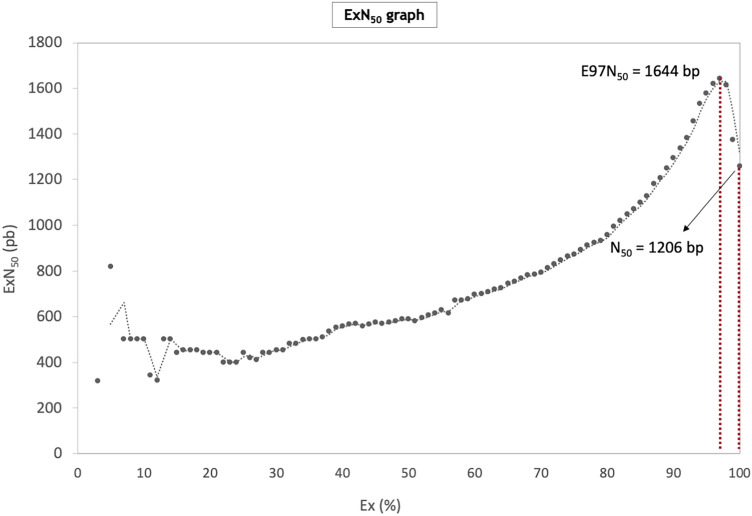
Expression-dependent N_50_ (ExN_50_), as calculated against a fraction of the total expressed data (Ex). ExN_50_ at the point of assembly saturation (97%) and traditional N_50_ are highlighted.

Trimmed reads were subjected to de novo whole transcriptome assembly using Trinity, after being further reduced to 73,014,522 by in silico normalization. Trinity assembler produced 691,300 transcripts, which were then clustered into 48,638 unigenes (i.e. uniquely assembled transcripts) with N_50_ 1206 bp. Based on BUSCO, the assembled transcriptome was highly complete with 94.9% ortholog genes from Eukaryota database being present with low fragmentation (2.7%), missing (2.4%), and duplication (15.7%). In addition, ExN_50_ statistics showed that the maximum N_50_ value was on E97% with 1644 bp N_50_ length (Fig. 1).

### Functional annotation

To evaluate the completeness of the transcriptome library, functional annotation—including GO terms, EggNOG and KEGG pathway enrichment analysis—of the whole transcriptome of *Tubastraea *spp. was performed using Blastx results and OmicsBox. A summary of the whole transcriptome assembly and annotation results is listed in Table 1.

**Table 1:** Summary of the transcriptome assembly, annotation, and differential abundance analysis.

**Total number of contigs**	75,006
Total number of unigenes	48,638
Blast annotated	59,505
InterProScan annotated	75,005
GO annotated	18,246
EggNOG annotated	44,319
KEGG annotated	4,203
**Differentially expressed genes FDR < 0.05, LogFC < ± 1**	4,483
Enriched genes in the total vs oral	3,174
Enriched genes in the oral vs total	1,309

### Differential expression analysis

**Figure 2:**
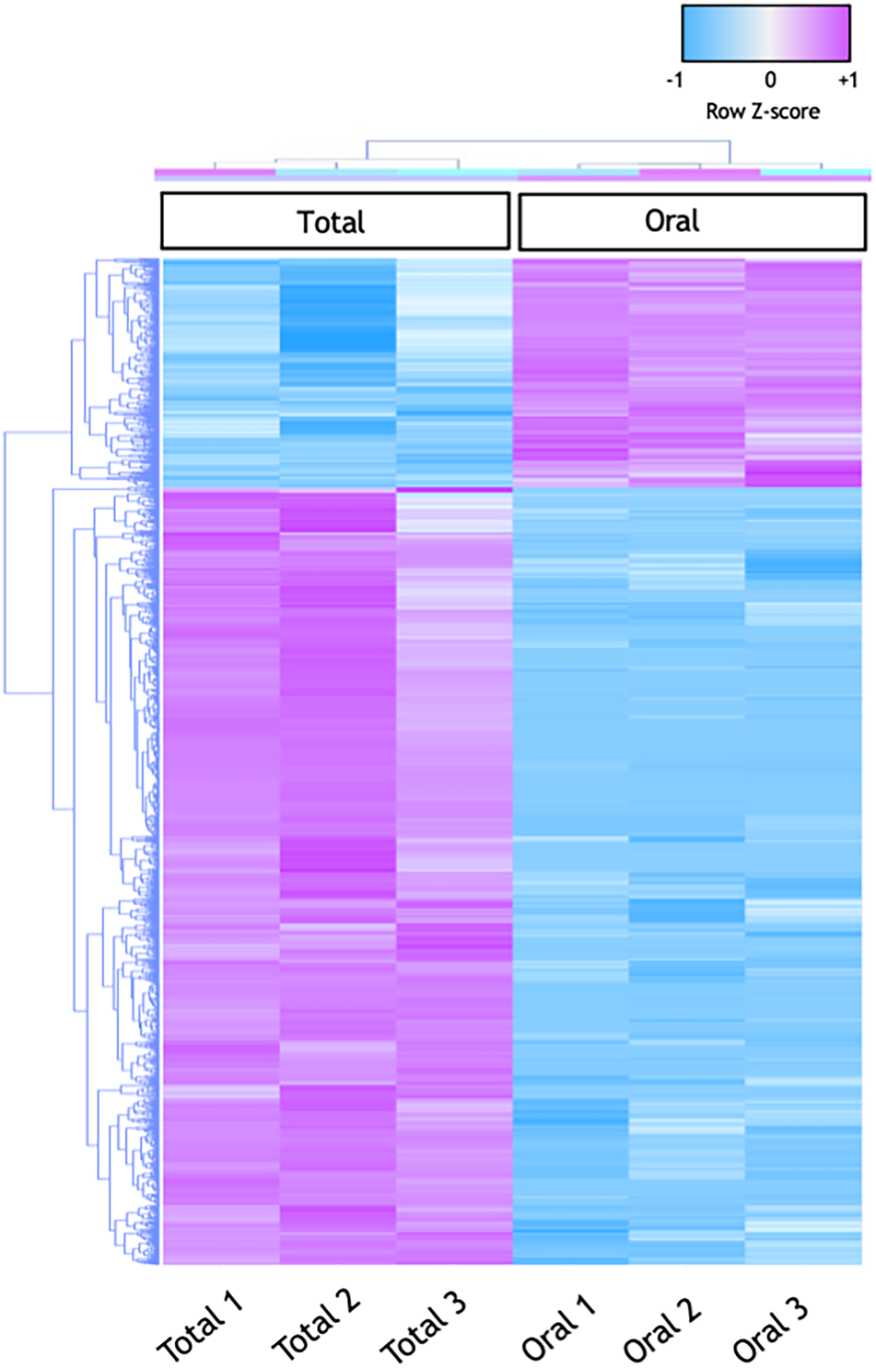
Heatmap and hierarchical cluster of 4,483 differentially expressed transcripts. The heatmap was generated using trimmed mean of M-values (TMM). Sample clustering was done using Pearson’s correlation. The Z-score scale is shown in the top-right corner ranging from − 1 (blue) to + 1 (violet).

**Figure 3:**
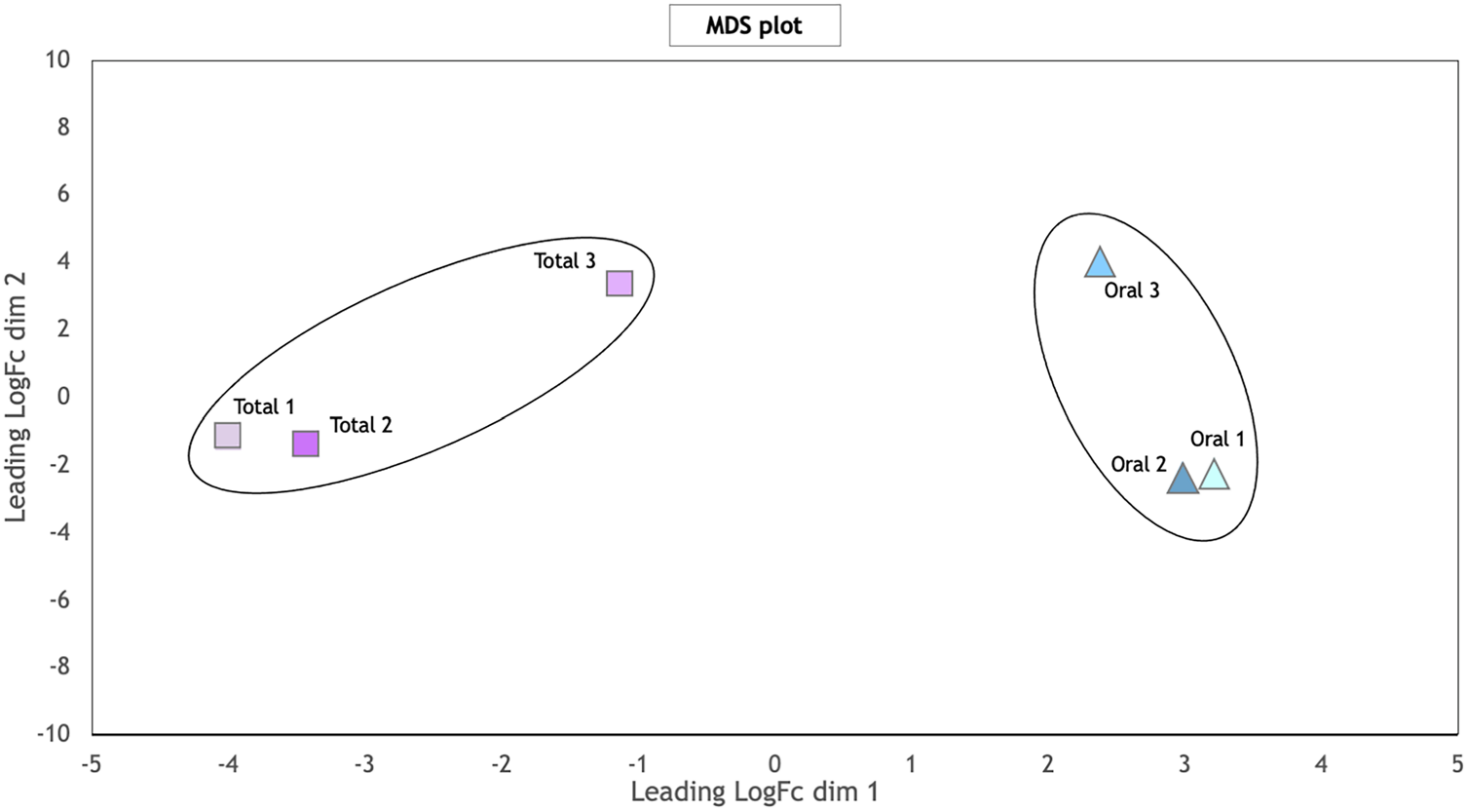
MDS plot of the data set. Samples (n = 6) are separated by the biological replicate in the first dimension and by the coral fraction in the second dimension.

To identify differentially expressed genes between the total colony and the oral fraction, we first selected genes that had count per millions (CPM) more than 1 in at least two samples. Differential expression analysis was then performed using OmicsBox, followed by Benjamini–Hochberg multiple test correction. A total of 4,483 genes were reported to be differentially expressed (FDR < 0.05, LogFC <  ± 1) between the total colony and the oral fraction (Table 1). Of these, 3,174 genes were significantly enriched in the total colony compared to the oral fraction, and 1,309 genes were significantly enriched in the oral fraction compared to the total colony. Differentially Expressed Genes (DEGs) have been clustered using Pearson’s correlation and displayed in a heatmap (Fig. 2). In this heatmap, biological replicates (1, 2 and 3) show strong clustering within the same group (Total and Oral), which are also clearly separated. In addition, a Multi-Dimensional Scaling (MDS) plot was performed to examine the homogeneity across biological replicates (Fig. 3). According to the MDS results, biological replicates showed strong clustering within each group and each group formed a distinct cluster.

### Comparison of calcification-related candidates between non-symbiotic and symbiotic scleractinian corals

**Figure 4:**
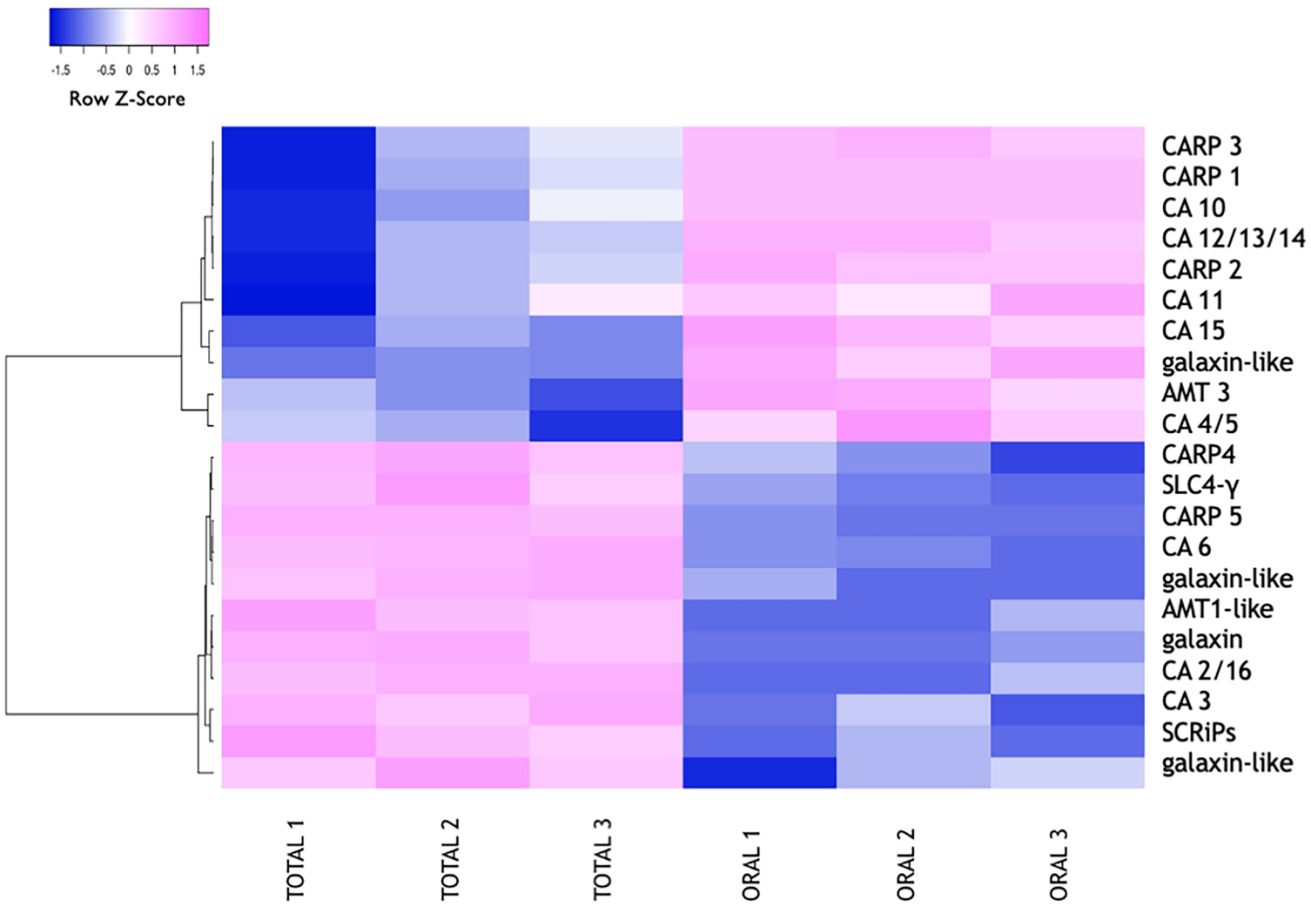
Heatmap of calcification-related genes differentially expressed between the total colony and the oral fraction.

To assess whether calcification-related candidates, previously identified in symbiotic scleractinian corals, were also present in *Tubastraea *spp., we performed a search for known homologs in the *Tubastraea *spp. transcriptome. Based on their role and their cellular/extracellular localization, these candidates can be divided into two groups: ion membrane transporters/enzymes and skeletal organic matrix proteins. Ion membrane transporters/enzymes comprise: Ammonium Transporters (AMTs)^46^, voltage gated proton channels (H_v_CNs)^47^, Na^+^/H^+^ exchanger (SLC9A)^47^, the SoLute Carrier 4-γ (SLC4-γ)^31^, Plasma Membrane Ca^2+^-ATPases (PMCAs)^30^ and the Voltage-Gated Ca^2+^-Channel (VGCC)^32^. Whereas, skeletal organic matrix proteins comprise: Coral Acid-Rich Proteins (CARPs)^35^, neurexins^48^, galaxins^49^ and Small Cysteine-Rich Proteins (SCRiPs)^50^. Carbonic Anhydrases (CAs)^34,51^ fall in between these two groups as they have been also identified in the organic matrix of the coral skeleton^13^. Our results show that, among the 75,006 contigs (Table 1), *Tubastraea *spp. possesses 55 protein sequences homologous to calcification-related candidates of symbiotic scleractinian corals (Table S1). Of these, only 21 (38%) are differentially expressed between the total colony and the oral fraction: 10/21 are enriched in the oral fraction compared to the total colony (CARP-1, -2 and -3, CA-4/5, 10, 11, 12/13/14 and 15, AMT3 and one galaxin-like) and 11/21 are enriched in the total colony compared to the oral fraction (CARP-4 and -5, SLC4-γ, CA-6, -2/16 and 3, two galaxin-like proteins and one galaxin, AMT1-like and SCRiPs) (Fig. 4 and Table S2). It is important to note that *Tubastraea *spp. CARPs and CAs have been named according to the phylogenetic trees provided in Figures S1 and S2.

Our results also show that 34 (62%) of the 55 protein sequences are not differentially expressed between the total colony and the oral fraction, and thus are not found in the heatmap (Fig. 4). These included: H_v_CNs, SLC9s, PMCAs, and VGCC.

### Functional annotation and identification of unigenes putatively involved in coral calcification

**Figure 5:**
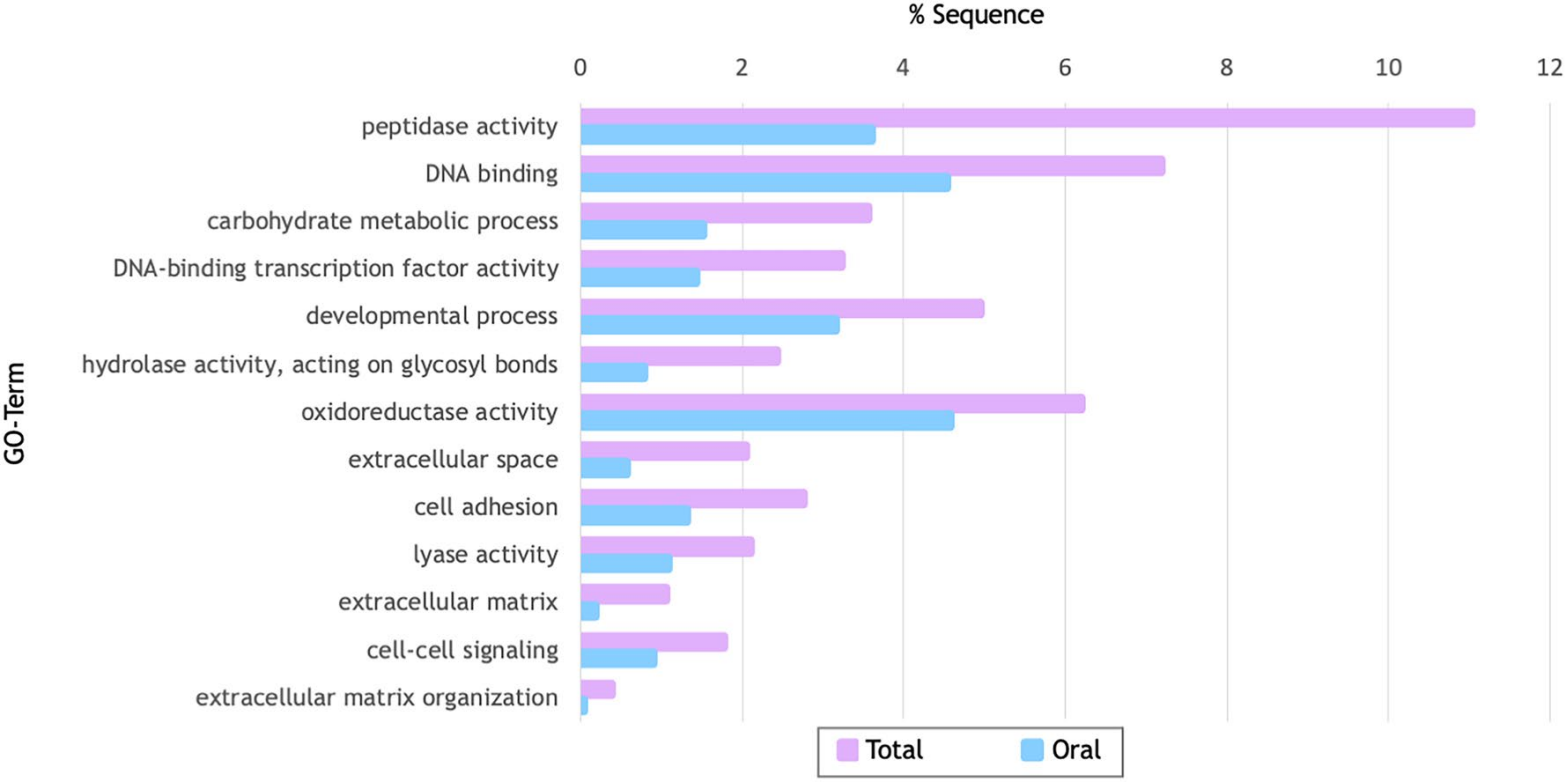
Gene Ontology Enrichment Analysis GO-terms associated with enriched genes in the total colony compared to the oral fraction.

DEGs were annotated using the same databases used for the whole transcriptome annotation. First, GO-term enrichment analysis using Fisher’s exact test was performed to infer which biological processes are associated with the enriched genes in the total colony compared to the oral fraction. Our results show 13 enriched GO-terms, including biological processes associated with “carbohydrate metabolic process”, “extracellular space”, “cell adhesion”, “extracellular matrix” and “extracellular matrix organization” (Fig. 5).

**Figure 6:**
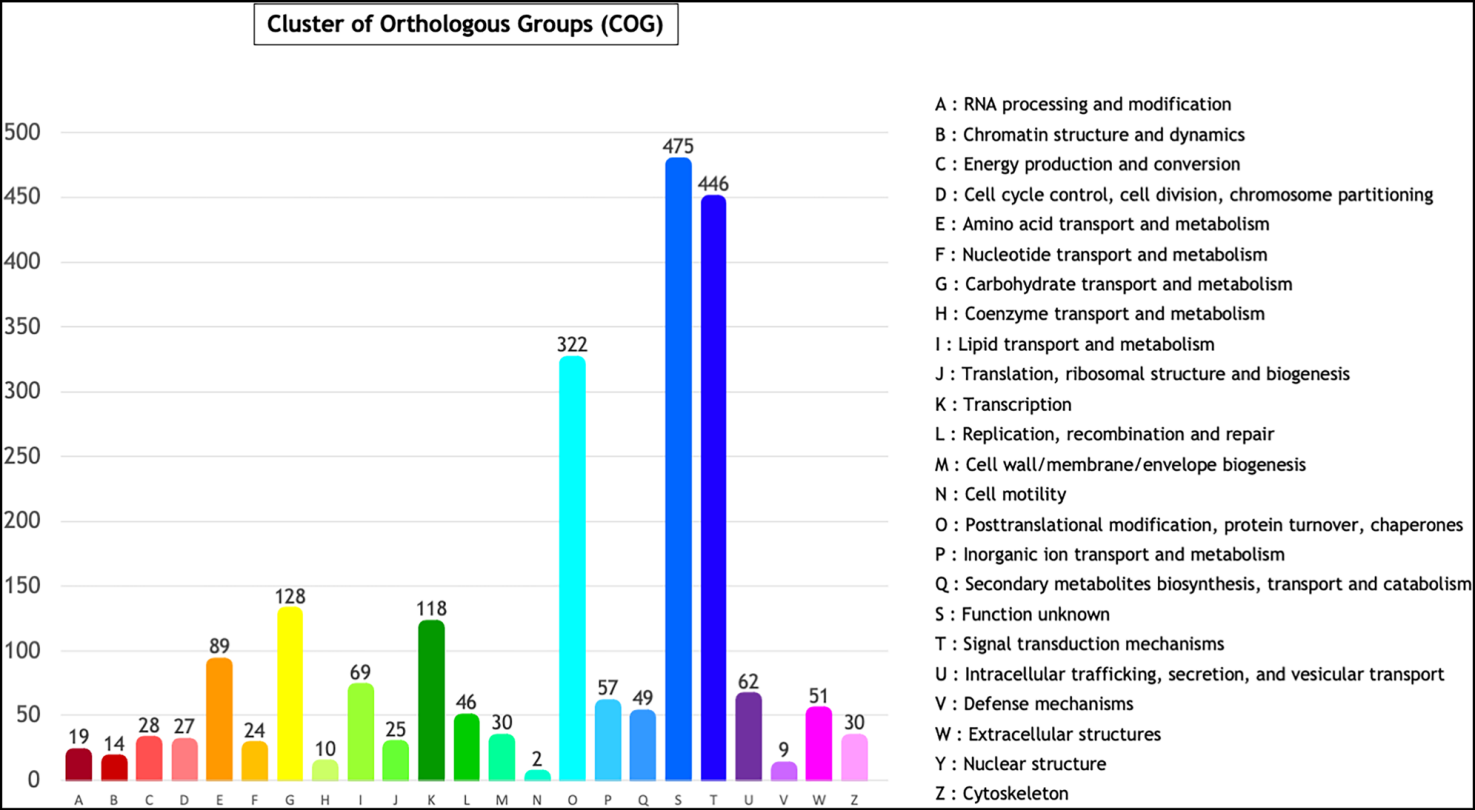
EggNOG classifications of enriched genes in the total colony compared to the oral fraction EggNOG categories are shown on the horizontal axis as alphabets with corresponding category names on the right.

EggNOG functional annotation of the enriched genes in the total colony compared to the oral fraction was then performed. A total of 2,841 out of 3,174 enriched genes (88.6%) are functionally annotated into 23 COG functional categories, including inorganic ion transport and metabolism (P), and intracellular trafficking, secretion and vesicular transport (U) (Fig. 6).

Finally, using the KO term, provided by the EggNOG mapper, of each annotated gene, we performed KEGG annotation. KEGG annotation further divided genes into multiple families. Among these, 39 KO terms are associated with ion transporters (Table 2).

**Table 2:** List of enriched transcripts, involved in ion transport, in the total colony compared to the oral fraction.

KO term	Ion transporters unigenes	Functional annotation	FC
K14611	TRINITY_DN1292_c0_g1_i21.p1	Solute carrier family 23 (nucleobase transporter), member 1	916.1
K15381	TRINITY_DN15243_c0_g1_i12.p1	MFS transporter, FLVCR family, disrupted in renal carcinoma protein 2	500.7
K05655	TRINITY_DN13741_c0_g1_i13.p1	ATP-binding cassette, subfamily B (MDR/TAP), member 8	333.1
K06580	TRINITY_DN11254_c0_g1_i6.p1	SLC42A; ammonium transporter Rh	317.7
K22190	TRINITY_DN43565_c0_g1_i3.p1	Membrane-spanning 4-domains subfamily A member 3/4/8/12/15/18	206.8
K21893	TRINITY_DN88858_c0_g1_i5.p1	Neuroglobin	182.3
K21396	TRINITY_DN29747_c0_g1_i6.p1	ATP-binding cassette, subfamily G (WHITE), eye pigment precursor transporter	100.7
K15104	TRINITY_DN18817_c0_g1_i1.p1	Solute carrier family 25 (mitochondrial oxoglutarate transporter), member 11	95.7
K21396	TRINITY_DN10851_c0_g1_i5.p1	ATP-binding cassette, subfamily G (WHITE), eye pigment precursor transporter	85.5
K08187	TRINITY_DN104999_c0_g1_i1.p1	Solute carrier family 16 (monocarboxylic acid transporters), member 10	62.1
K17769	TRINITY_DN45389_c0_g1_i4.p1	Mitochondrial import receptor subunit TOM22	61.4
K13868	TRINITY_DN7767_c0_g1_i15.p1	Solute carrier family 7 (L-type amino acid transporter), member 9/15	53.2
K14387	TRINITY_DN6094_c0_g1_i13.p1	Solute carrier family 5 (high affinity choline transporter), member 7	52.7
K14613	TRINITY_DN38892_c0_g1_i1.p1	Solute carrier family 46 (folate transporter), member 1	49.4
K09866	TRINITY_DN9220_c0_g1_i2.p1	Aquaporin-4	43.4
K08204	TRINITY_DN84629_c0_g1_i1.p1	Solute carrier family 22 (organic anion transporter), member 7	42.5
K05673	TRINITY_DN43332_c0_g1_i2.p1	ATP-binding cassette, subfamily C (CFTR/MRP), member 4	39.9
K08187	TRINITY_DN57711_c0_g1_i2.p1	Solute carrier family 16 (monocarboxylic acid transporters), member 10	39.2
K15109	TRINITY_DN17474_c0_g1_i7.p1	Solute carrier family 25 (mitochondrial carnitine/acylcarnitine transporter), member 20/29	35.1
K15108	TRINITY_DN12359_c10_g1_i3.p1	Solute carrier family 25 (mitochondrial thiamine pyrophosphate transporter), member 19	32.2
K05643	TRINITY_DN170738_c0_g1_i2.p1	ATP-binding cassette, subfamily A (ABC1), member 3	23.7
K14445	TRINITY_DN38284_c0_g1_i1.p1	Solute carrier family 13 (sodium-dependent dicarboxylate transporter), member 2/3/5	16.4
K05673	TRINITY_DN29216_c2_g1_i3.p1	ATP-binding cassette, subfamily C (CFTR/MRP), member 4	13.6
K21396	TRINITY_DN29747_c0_g1_i3.p1	ATP-binding cassette, subfamily G (WHITE), eye pigment precursor transporter	12.4
K05673	TRINITY_DN73454_c0_g1_i2.p1	ATP-binding cassette, subfamily C (CFTR/MRP), member 4	8.9
K08204	TRINITY_DN23500_c0_g1_i4.p1	Solute carrier family 22 (organic anion transporter), member 7	8.8
K14686	TRINITY_DN90716_c0_g1_i1.p1	Solute carrier family 31 (copper transporter), member 1	8.6
K21862	TRINITY_DN29586_c0_g1_i7.p1	Voltage-gated cation channel	8.5
K21988	TRINITY_DN5910_c4_g1_i1.p1	Transmembrane channel-like protein	7.4
K14611	TRINITY_DN1292_c0_g1_i23.p1	Solute carrier family 23 (nucleobase transporter), member 1	6.8
K08204	TRINITY_DN23500_c0_g1_i3.p1	Solute carrier family 22 (organic anion transporter), member 7	6.5
K14445	TRINITY_DN20897_c0_g1_i1.p1	Solute carrier family 13 (sodium-dependent dicarboxylate transporter), member 2/3/5	6.4
K15109	TRINITY_DN22386_c0_g1_i6.p1	Solute carrier family 25 (mitochondrial carnitine/acylcarnitine transporter), member 20/29	6.4
K14994	TRINITY_DN9280_c0_g1_i8.p1	Solute carrier family 38 (sodium-coupled neutral amino acid transporter), member 7/8	6.3
K05039	TRINITY_DN7450_c0_g1_i13.p1	Solute carrier family 6, member 6	5.7
K15015	TRINITY_DN68697_c0_g1_i1.p1	Solute carrier family 32 (vesicular inhibitory amino acid transporter)	5.7
K08187	TRINITY_DN26257_c3_g1_i4.p1	Solute carrier family 16 (monocarboxylic acid transporters), member 10	5.3
K11536	TRINITY_DN3340_c0_g2_i6.p1	Pyrimidine nucleoside transport protein	5.3
K05038	TRINITY_DN12243_c1_g1_i1.p1	Solute carrier family 6, member 5/9	5.1
K00799	TRINITY_DN12289_c0_g1_i14.p1	Glutathione S-transferase	4.8
K21862	TRINITY_DN9832_c0_g1_i2.p1	Voltage-gated cation channel	4.6
K08204	TRINITY_DN19075_c0_g1_i1.p1	Solute carrier family 22 (organic anion transporter), member 7	4.3
K08187	TRINITY_DN16679_c0_g2_i1.p1	Solute carrier family 16 (monocarboxylic acid transporters), member 10	4.2
K14453	TRINITY_DN16687_c0_g1_i7.p1	Solute carrier family 26	4.2
K14683	TRINITY_DN5423_c0_g1_i6.p1	Solute carrier family 34 (sodium-dependent phosphate cotransporter)	4.2
K03320	TRINITY_DN16401_c0_g1_i7.p1	Ammonium transporter, Amt family	4.1
K05048	TRINITY_DN609_c1_g1_i13.p1	Solute carrier family 6, member 15/16/17/18/20	3.9
K21396	TRINITY_DN10851_c0_g1_i4.p1	ATP-binding cassette, subfamily G (WHITE), eye pigment precursor transporter	3.8
K05048	TRINITY_DN609_c1_g1_i14.p1	Solute carrier family 6, member 15/16/17/18/20	3.6
K05678	TRINITY_DN8486_c0_g1_i4.p1	ATP-binding cassette, subfamily D (ALD), member 4	3.5
K05048	TRINITY_DN2910_c0_g1_i4.p1	Solute carrier family 6, member 15/16/17/18/20	3.3
K00799	TRINITY_DN1669_c0_g1_i3.p1	Glutathione S-transferase	3.2
K15281	TRINITY_DN9775_c0_g1_i5.p1	Solute carrier family 35	3.2
K14995	TRINITY_DN19648_c0_g1_i1.p1	Solute carrier family 38 (sodium-coupled neutral amino acid transporter), member 9	3.2
K06580	TRINITY_DN1639_c0_g1_i6.p1	SLC42A; ammonium transporter Rh	3.1
K12385	TRINITY_DN8972_c0_g1_i9.p1	Niemann-Pick C1 protein	3.1
K05038	TRINITY_DN8948_c0_g1_i15.p1	Solute carrier family 6, member 5/9	2.9
K15015	TRINITY_DN14356_c0_g1_i5.p1	Solute carrier family 32 (vesicular inhibitory amino acid transporter)	2.9
K15381	TRINITY_DN1196_c2_g2_i3.p1	MFS transporter, FLVCR family, disrupted in renal carcinoma protein 2	2.9
K11518	TRINITY_DN2445_c7_g1_i4.p1	Mitochondrial import receptor subunit TOM40	2.8
K14613	TRINITY_DN9535_c0_g1_i2.p1	Solute carrier family 46 (folate transporter), member 1	2.7
K12385	TRINITY_DN2483_c0_g1_i6.p1	Niemann-Pick C1 protein	2.5
K05643	TRINITY_DN2705_c1_g1_i6.p1	ATP-binding cassette, subfamily A (ABC1), member 3	2.3
K05399	TRINITY_DN12573_c1_g1_i4.p1	Lipopolysaccharide-binding protein	2.1

## Discussion

The “calcification toolkit” is the collective term documented and/or hypothesized to be involved in biomineral formation at various stages of an organism’s life history^52^. Out of all the toolkit components, proteins have been the most intensively characterized^26,53,54^. As a result, proteomic studies have suggested that, although proteins from distant organisms share common properties^53^, each taxon-specific suite appears to have evolved independently through convergent and co-option evolution. This has led to variable contributions, from new lineage- and species-specific proteins, to the “calcification toolkit”, which show contrasting rates of conservation between and within lineages^55^. Several tools of the “calcification toolkit” have also been identified in scleractinian corals^48,56^, yet to date only few experiments have been conducted and solely for symbiotic species^54^. Other than being particularly attractive for calcification studies because of the lack of symbiotic dinoflagellates in their tissues, corals belonging to the *Tubastraea* genus have been the focus of numerous biological^57–59^ and ecological research studies^60,61^ aiming at identifying key parameters underlying their invasiveness. Nevertheless, their “calcification toolkit”, which may include specific components providing these corals with an advantage in terms of calcification strategies, has never been investigated at the molecular level. In this study, we aimed to fill this knowledge gap by searching for candidates of the “calcification toolkit” in the non-symbiotic scleractinian coral *Tubastraea *spp. using a tissue micro-dissection technique to remove the oral fraction (easily accessible and free of the calicoblastic cells) from the total colony of *Tubastraea *spp. This previously developed technique has already been used in the past and has contributed to the identification of some of the most frequently searched and studied candidates in a wide range of calcifying metazoans^26,62,63^, other than corals^31,45,47^. By coupling this technique with RNA-seq, we have then identified and analyzed differentially expressed genes with a focus on those enriched in the total colony compared to the oral fraction. Indeed, these genes are specific of the aboral tissues and include calicoblastic cell-specific genes, that could play a role in calcification. This is supported by our results showing that, although many genes are ubiquitously expressed in the total colony—and thus in both oral and aboral tissues -, others are differentially expressed, with clearly distinct expression profiles between the total colony and the oral fraction (Figs. 2 and 3). It follows that the different expression profiles reflect specific gene functions related to the oral and aboral tissues. Amongst the 3,174 aboral-specific genes (Table 1), we identified most calcification-related candidates previously described as part of the “calcification toolkit” of symbiotic scleractinian corals (Fig. 4). These include the bicarbonate transporter SLC4-γ^64^. SLC4-γ has been proposed to play a role both in the regulation of intracellular HCO_3_^-^ homeostasis—which is critical to buffer excess of H^+^ generated during CaCO_3_ precipitation—and the supply of HCO_3_^-^ to the calcifying cells in several organisms, including sea urchins^63,65^, mussel^62^, coccolithophores^66^ and corals^31,67^. We also identified an ammonium transporter belonging to the AMT1 sub-clade (Fig. 4). AMT transporters have been suggested to play a role in calcification in multiple metazoans, including mollusks^68–70^ and symbiotic scleractinian corals^71–73^. Although their role in coral calcification still needs to be investigated in detail, it has been suggested that AMT1 transporters mediate pH regulation in the ECM by transporting NH_3_ into the ECM which could buffer excess of protons. Organic matrix proteins, including 2 CARPs and 1 SCRiP, were also identified (Fig. 4). CARPs are proteins with dominant Low Complexity Domains (LCDs) that have been described in the secreted organic matrix of biominerals in different metazoan taxa^74–77^. CARPs have been identified also in previous proteomic studies on coral skeletons^35^, where they have been suggested to play a role in CaCO_3_ formation given their high affinity to positively charged ions (i.e. Ca^2+^)^78–80^. SCRiPs, instead, are a family of putatively coral-specific genes for which different roles have been suggested based on their molecular features (i.e. presence of signal peptide, high amino acidic residues content and cysteine-rich)^50^. Moreover, three galaxins-like proteins and three CAs were also identified (Fig. 4). Galaxin was first identified in the exoskeleton of the scleractinian coral *Galaxea fascicularis* and was described as a tandem repeat structure with a di-cysteine motif fixed at nine positions^49^. Since this discovery, galaxin homologs have been observed in the exoskeleton of other scleractinian species^81–83^, as well as in mollusks^84^ and squid^85^. It has also been shown that galaxin is associated with the developmental onset of calcification after larval stage in *Acropora millepora*^86^. Whereas CAs are metallo-enzymes that catalyze the reversible hydration of CO_2_ into HCO_3_^-^, the source of inorganic carbon for CaCO_3_ precipitation. In metazoans, CAs belong to a multigenic family and are widely known to be involved in calcification in diverse metazoans such as sponge spicules^87,88^, mollusk shells^89,90^, sea urchin skeleton^91^, and bird eggshells^92^, as well as scleractinian corals^34,51^. In *Tubastraea aurea*, CAs were previously identified both in the coral tissues and the skeletal organic matrix, where they have been suggested to play a direct role in calcification^13^. Here, we have identified 3 CAs with higher expression in the total colony compared to the oral fraction, which suggests a potential role in calcification.

The presence of these candidates amongst the aboral-specific genes of a non-symbiotic scleractinian coral strongly suggests a calcification-related function and, in a wider context, further supports the hypothesis of a “common calcification toolbox” in scleractinian corals, as previously suggested^93^.

However, several components of the toolbox have not been identified amongst the aboral-specific genes of *Tubastraea *spp. (Fig. 4). These include: (1) voltage-gated H^+^ channels (H_v_CN), that have been suggested to participate in the pH_i_ homeostasis of calcifying coccolithophore cells^94^, in the larval development and shell formation of the blue mussel^62^ and in the calicoblastic cells of several symbiotic scleractinian coral species^47,95^; (2) SLC9s, that have been suggested to play a role in H^+^ removal during trochophore development in mussels^62^ and coral calcification^47^, (3) Plasma Membrane Ca^2+^-ATPase (PMCA), which have been suggested to take part in Ca^2+^ supply to the sites of calcification in mussels^62^, as well as in the pH_ECM_ regulation of the ECM in corals^30^, neurexins, that connect the calicoblastic cells to the extracellular matrix in corals^48^ and (4) Voltage-Gated Ca^2+^-channels (VGCC), which have been suggested to facilitate Ca^2+^ transport in the calcifying epithelium of oysters^96^ and corals^30^. One possible explanation of these results is that gene gain/loss or even change of protein function has occurred during scleractinian evolutionary history, resulting in a different “calcification toolkit”. This is further supported by the hypothesis that, although calcification-related proteins from distant organisms share common properties^53^, they have evolved independently, through convergent evolution and co-option, in each taxon, thus resulting in contrasting rates of conservation between and within lineages^55^.

In addition to comparing these calcification-related candidates between non-symbiotic and symbiotic scleractinian corals, we have also searched for novel ion transporter candidates of the “calcification toolkit” in *Tubastraea *spp. by focusing on the rest of the aboral-specific genes. To explore their involvement in biological processes, we performed a GO enrichment analysis, and showed that, among several processes, these aboral-specific genes are enriched in “extracellular space”, “cell adhesion”, “extracellular matrix” and “extracellular matrix organization” (Fig. 5). These results highlight the importance of extracellular matrices in the aboral tissues, in which they play a pivotal role in the spatial organization of the cells, as they organize them according to their function. Some examples include both the organic extracellular matrix (ECM), which facilitates cell–cell and cell-substrate adhesion with the help of desmocytes^97^, as well as the skeletal organic matrix (SOM), which facilitates the controlled deposition of the CaCO_3_ skeleton. Also, the expression of genes in the aboral tissues linked to “carbohydrate metabolic processes” suggests an enrichment of biochemical processes involved in carbohydrate metabolism, which may ensure a constant supply of energy needed to support the energy-demanding process of calcification^98,99^.

Moreover, EggNOG annotation (Fig. 6) shows that some of the aboral-specific genes belong to the following categories: “inorganic ion transport and metabolism” and “intracellular trafficking, secretion, and vesicular transport”, thus underling the importance of membrane and vesicular transport linked to calcification in the aboral tissues^2,14,18^. These results are supported by recent observations of intracellular vesicles moving towards the calcification site both in corals^14,18^ and sea urchins^100^. Calcification-related ions have been suggested to be highly concentrated in these vesicles in order to promote the formation of ACC nanoparticles, which are successively deposited into the calcification compartment where crystallization occurs^14^. The regulation of endocytosis and vesicular transport between membrane-bound cellular compartments is therefore strictly necessary in coral calcification, and the identification of genes related to these pathways, among the *Tubastraea *spp. aboral-specific genes, further underlines their importance also in non-symbiotic scleractinian species.

KEGG analysis further allowed us to identify a list of candidates that could play a role in calcification related ion transport (Table 2). The list includes several genes belonging to the ammonium transporter family (AMT/Rh/MEP), notably, AMT and Rh homologs. As well as for AMT transporters, also Rh transporters have been suggested to be involved in coral calcification. Rh homologs have been identified in the calicoblastic epithelium of the symbiotic scleractinian coral *Acropora yongei*, where they have been suggested to mediate a possible pathway for CO_2_—a critical substrate for CaCO_3_ formation—in the ECM^101^. The identification of these genes also among the *Tubastraea *spp. aboral-specific ones strongly suggests a direct role of these transporters in non-symbiotic scleractinian calcification.

We also identified a large number of transporters belonging to the SoLute Carrier (SLC) families that, in vertebrates, constitute a major fraction of transport-related genes ^102^ (Table 2). Some of these members (SLC7, SLC25 and SLC35) have been previously reported to be involved in coral thermal stress, while others (SLC26) have been proposed to participate in coral larval development^103^, as well as cellular pH and bicarbonate metabolism^31^.

Two plasma-membrane homologs belonging to the SLC13 family have also been identified (Table 2). These transporters function as Na^+^-coupled transporters for a wide range of tricarboxylic acid (TCA) cycle intermediates^104^, and have been widely described in vertebrates for their role in calcification^105–108^. The tricarboxylic acid citrate has also been found to be strongly bound to the bone nanocrystals in fish, avian, and mammalian bone^109^, whereas in corals no study has shown the presence of citrate in the skeleton. In invertebrates, SLC13 members have been mainly described for their role in nutrient absorption^110,111^, as they provide TCA cycle metabolites, that are used for the biosynthesis of macromolecules, such as lipids and proteins^2,20^. These macromolecules are among the principal components of the skeletal organic matrix and SLC13 members might contribute to their transport into the coral aboral tissues.

SLC16 family members, and precisely the monocarboxylic acid transporters (MCTs), are also enriched in the total colony compared to the oral fraction (Table 2). Members of the SLC16 family comprise several subfamilies that differ in their substrate selectivity^112^. In corals and sea anemones, SLC16 subfamilies transporting aromatic amino acids have been mostly characterized for their role in nutrient exchange between the coral host and its symbionts^36,113^, while no information is available for those transporting monocarboxylic acids. In human, MCTs function as pH_i_ regulatory transporters by mediating the efflux of monocarboxylic acid (predominantly lactate) and H^+^, in tissues undergoing elevated anaerobic metabolic rates^114,115^, and in *Tubastraea *spp. they might be involved in H^+^ extrusion at the sites of calcification perhaps functioning as pH_i_ regulators.

Interestingly, members of the SLC23 family (FC = 916.1), which comprise ascorbic acid transporters, are the most enriched in the total colony compared to the oral fraction, in *Tubastraea *spp. (Table 2). This result is in agreement with another RNA-seq study performed on swimming and settled larvae of the coral *Porites astreoides*, also showing that an SLC23 transporter is among the most highly expressed ion transporters in larvae initiating calcification^116^. Ascorbic acid is an essential enzyme cofactor that participates in a variety of biochemical processes, most notably collagen synthesis^117,118^. Collagen is a fibrillar protein, that forms one of the main components of extracellular matrices^119^. Previous studies have shown that the addition of ascorbic acid stimulates collagen production in many metazoans^120–122^, including corals^119^. It is thus possible that SLC23 transporters might provide ascorbic acid to the aboral tissues, and potentially the calcifying cells, which use it to promote collagen that, together with other ECM proteins, builds a structural framework for the recruitment of calcium binding proteins, as previously suggested^48,123–125^.

Last but not least, our study also identified so-called “dark genes”, i.e., genes that lack annotation^126^, within the list of aboral-specific genes. These genes are potentially equally important, as they are expressed in the aboral tissues along with other genes with known functions in calcification. It is therefore possible that “dark genes” and calcification-related genes may be linked, as they can be involved in the same pathway e. g. enzymes and/or regulatory factors. Heterologous expression of “dark genes” in model systems, easy to manipulate and with available molecular tools for visualizing gene expression and protein localization (e. g. *Nematostella vectensis*) could be taken in consideration to investigate their role and contribute to functional annotation^127^.

## Conclusion

**Figure 7:**
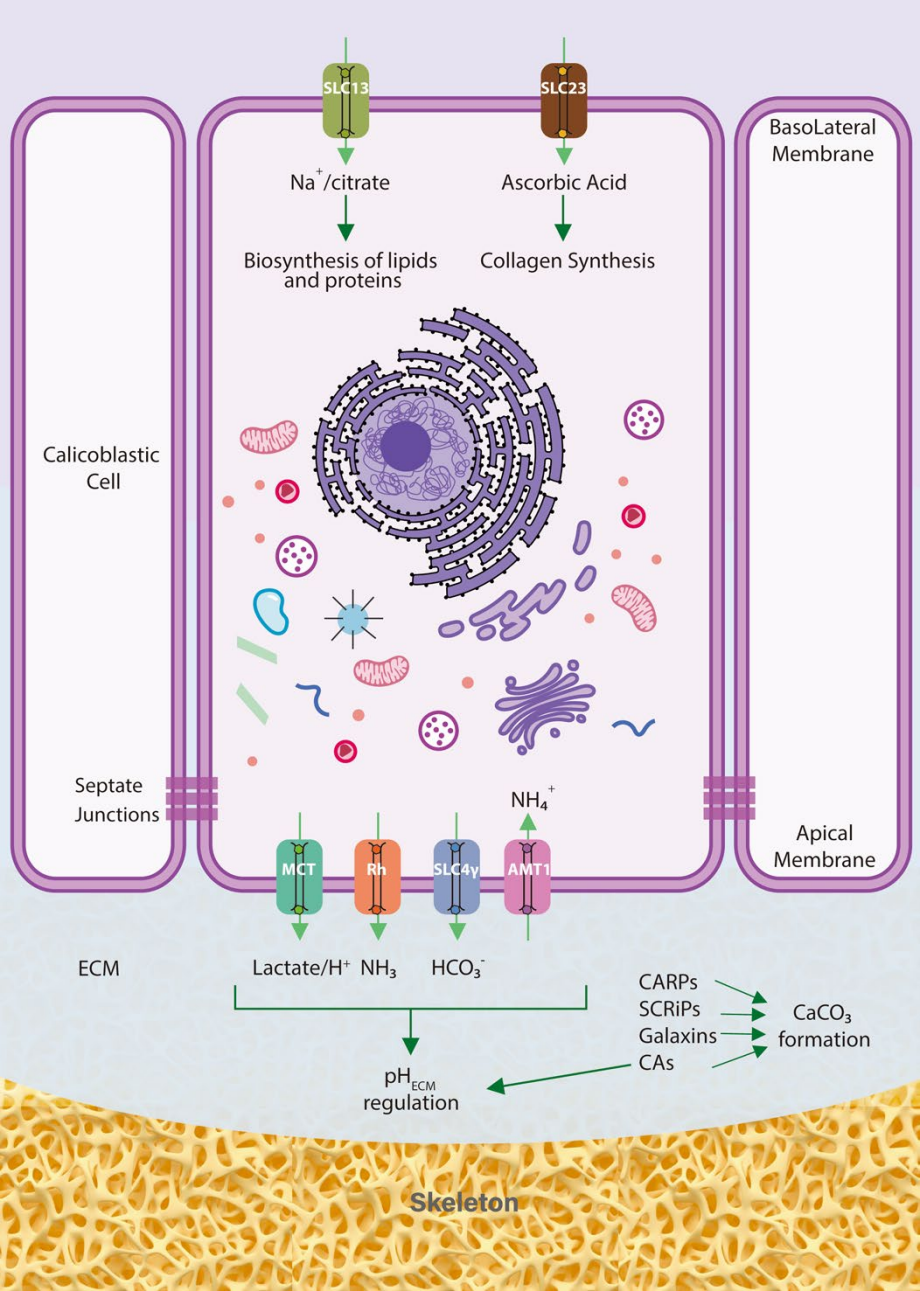
Model of a calicoblastic cell from a non-symbiotic scleractinian coral showing aboral specific ion transporter candidates identified in this study. Cellular localization of ion transporters on the apical and basolateral membrane is hypothetical and has been assigned based on previous immunolocalization studies and/or existing literature in other model systems. Dark green arrows indicate putative biological processes in which ion transporters could be involved. Abbreviation: ECM: Extracellular Calcifying Medium.

The *Tubastraea *spp. transcriptome here provided is a fundamental tool which promises to provide insights not only about the genetic basis for the extreme invasiveness of this particular coral genus, but also to understand the differences between calcification strategies adopted by symbiotic and non-symbiotic scleractinian corals at the molecular level. The analysis of the aboral-specific genes of *Tubastraea *spp. revealed numerous candidates for a potential role in scleractinian calcification, including both previously described candidates (SLC4-γ, AMT-1like) and novel ion transporters (SLC13, −16, −23, and others) (Fig. 7). Future studies will then be required to better dissect the precise mechanisms behind these candidates and may offer further knowledge which could lead to the development of novel biotechnological strategies for prevention, management, and control of this and other invasive species.

## Methods

### Biological material and experimental design

Experiments were conducted on non-symbiotic corals belonging to the *Tubastraea* genus*.* Corals belonging to this genus possess poorly defined taxonomic features and several unidentified morphotypes that severely challenge species identification^128^. Three independent *Tubastraea *spp. colonies, of unknown genotype, were collected in Australia in February 2018. The colonies were purchased from De JONG Marine Life, Netherland and imported with the CITES N°2018MC39519. These were grown in the long-term culture facilities at the Centre Scientifique de Monaco in aquaria supplied with seawater from the Mediterranean Sea (exchange rate 2% h^−1^ and flow rate of 20 L/h), under the following controlled conditions: semi-open circuit, temperature of 26 °C and no light. Corals were fed daily with frozen rotifers and twice a week with live *Artemia salina nauplii*.

### Micro-dissection, RNA isolation and sequencing from *Tubastraea *spp.

Three biological replicates of *Tubastraea *spp. were micro-dissected by separating the oral fraction from the total colony. Then, RNA was extracted from each fraction, as previously described^47^. Preparation of mRNAs, fragmentation, cDNA synthesis, library preparation, and sequencing using Illumina HiSeq™ 2000 were performed at the King Abdullah University of Science and Technology (KAUST)^93^.

### Data analysis pipeline

Data analysis pipeline contained three major sections including: raw data pre-processing, de novo transcriptome assembly and post-processing of the transcriptome. First, raw reads of six individual libraries were subjected to quality trimming, using the software Trimmomatic (version 0.36)^129^. This step consisted in trimming low quality bases, removing N nucleotides, and discarding reads below 36 bases long. Then, contaminant sequences were removed, using the software BBDuk^130^, by blasting raw reads against a previously created contaminant_DB of the most common contaminant species—including Symbiodiniaceae. Clean and trimmed reads from all samples were then pooled together and further assembled using Trinity software (version 2.8.0) with default parameters^131^. The *in-silico* normalization was performed within Trinity prior to de novo assembly. To obtain sets of non-redundant transcripts, we applied the following filtering steps: (1) transcripts with more than 95% of identity were clustered together using CD-HIT software^132^ and (2) all likely coding regions were filtered by selecting the single best open reading frame (ORF) per transcript, using TransDecoder (version 3.0.0)^133^. Also, in the latter step, transcripts with ORFs < 100 base pairs (bp) in length were removed before performing further analyses. The final transcriptome (referred to as transcriptome_all) was subjected to quality assessment via generation of ExN50 statistics, using “contig_ExN50_statistic.pl”, and examination of orthologs completeness, using BUSCO (version 3) against eukaryota_odb10 database^134^. Transcriptome_all was then aligned against NCBI’s non-redundant metazoan databases using Blastx^135^, with a cutoff E-value of < 10^–15^, and the alignment results were used to annotate all the unigenes (= uniquely assembled transcripts). For their further annotation and classification, OmicsBox software (version 2.0.36)^136^ was used to assign Gene Ontology (GO) terms^137^, Evolutionary Genealogy of Genes: Non-supervised Orthologous Groups (EggNOG)^138^ and Kyoto Encyclopedia of Genes and Genomes (KEGG) pathway enrichment analysis^139^. Additionally, differential abundance analysis to identify differentially expressed genes (DEGs) was performed using OmicsBox^136^. To convert the RNA-Seq data into quantitative measure of gene expression, we calculated the number of RNA-Seq reads mapping to transcriptome_all. Transcripts that had at least a log fold change (LogFC) of ± 1 with a false discovery rate (FDR or adjusted *p*-value) less than 0.05 were considered as differentially expressed.

## Supplementary Information

Supplementary Information 1.

Supplementary Information 2.

Supplementary Information 3.

Supplementary Information 4.

Supplementary Information 5.

## Data Availability

All data needed to evaluate the conclusions in the paper are present in the manuscript and/or the Additional Files. Additional data related to this manuscript may be requested from the authors. Genomic and transcriptomic data were obtained from the public available database of the National Center for Biotechnology Information or from the private database of the Centre Scientifique de Monaco.
