# Supplementary figures and images for: Investigating calcification-related candidates in a non-symbiotic scleractinian coral, Tubastraea spp
Source: Sci Rep. 2022 Aug 6;12:13515. doi: 10.1038/s41598-022-17022-4 (PMC9357087; doi:10.1038/s41598-022-17022-4)

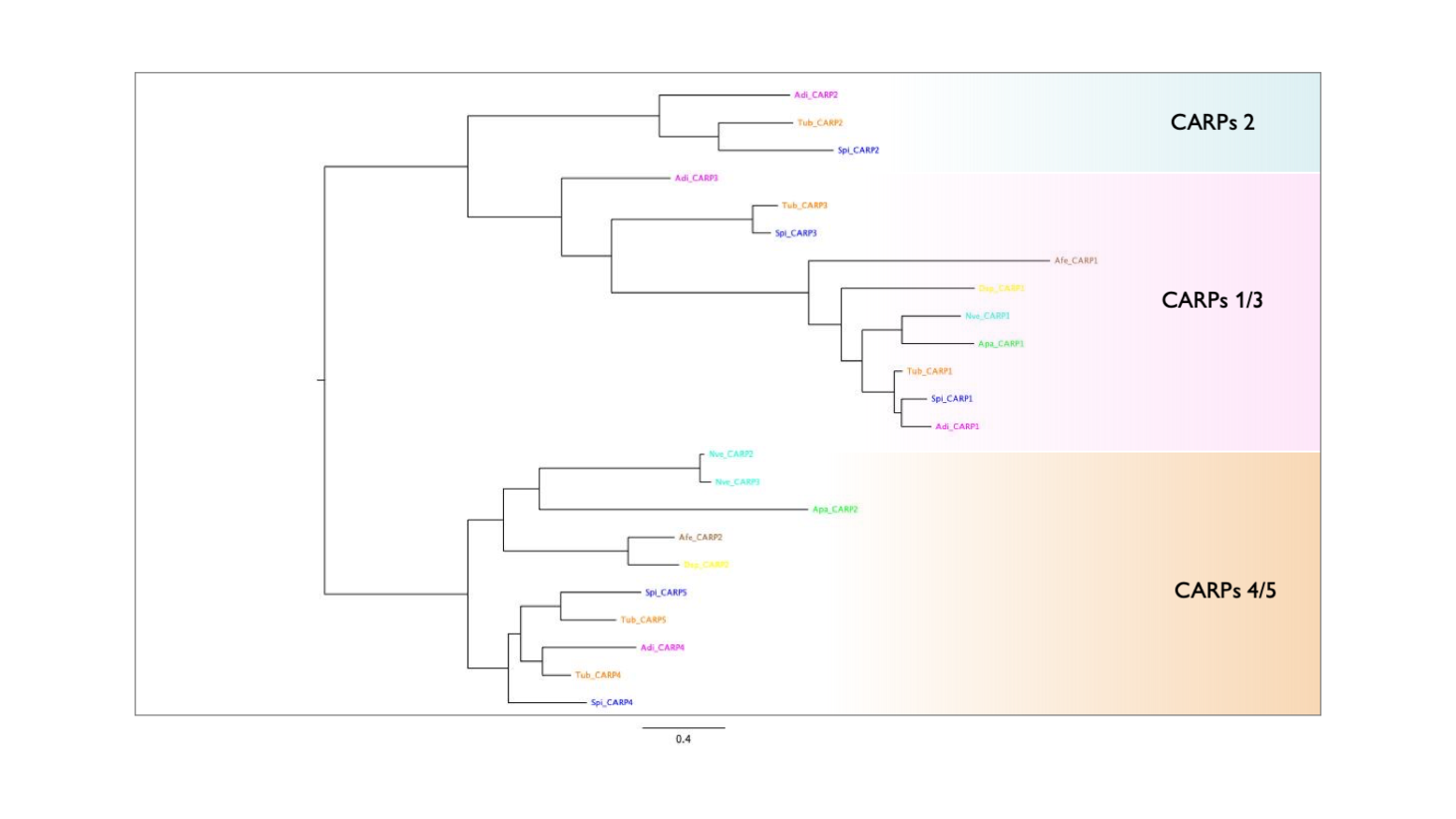

Supplement: Supplementary file 1 — Supplementary Information 1. [file 41598_2022_17022_MOESM1_ESM.tiff]

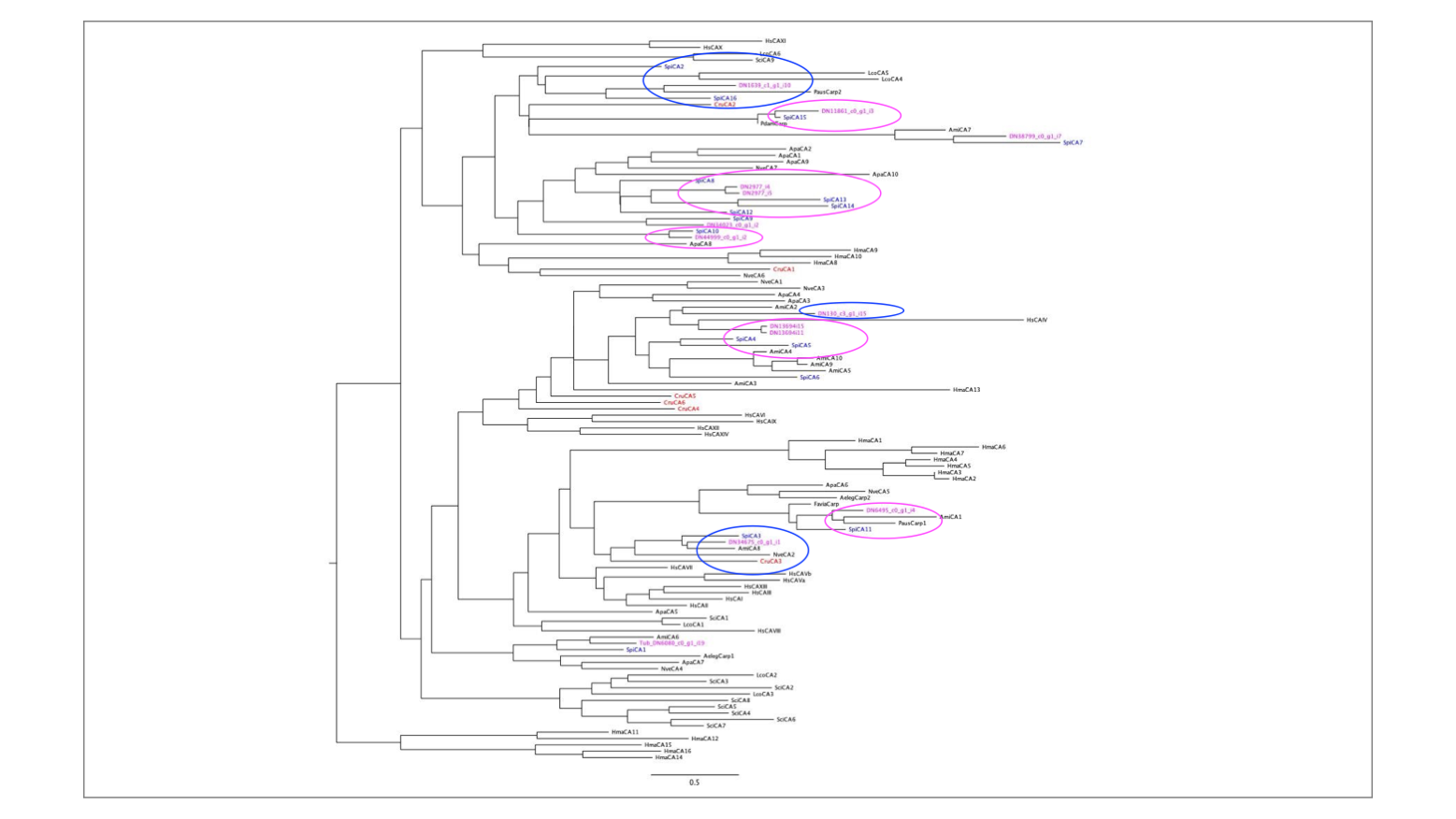

Supplement: Supplementary file 2 — Supplementary Information 2. [file 41598_2022_17022_MOESM2_ESM.tiff]

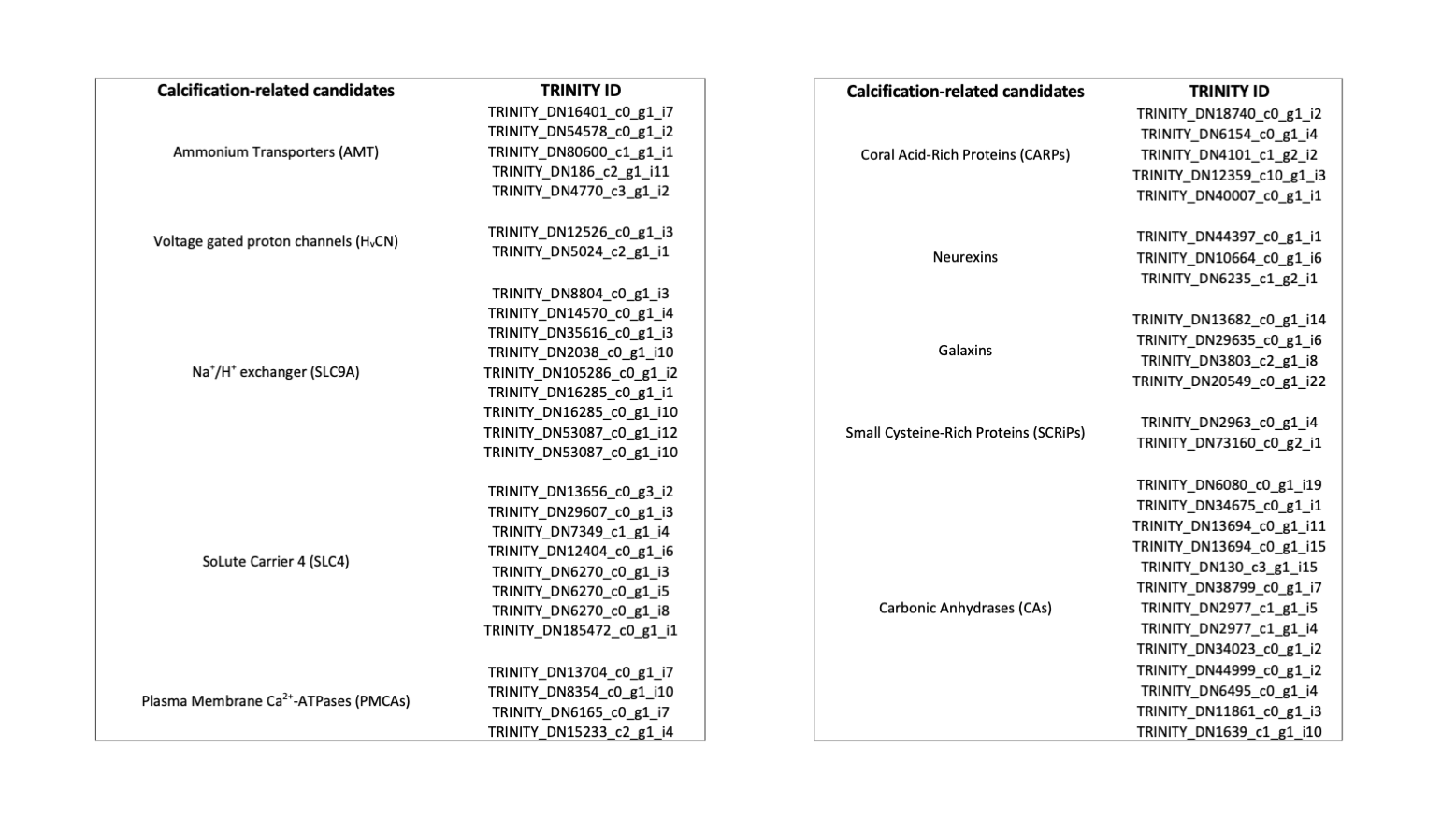

Supplement: Supplementary file 4 — Supplementary Information 4. [file 41598_2022_17022_MOESM4_ESM.tiff]

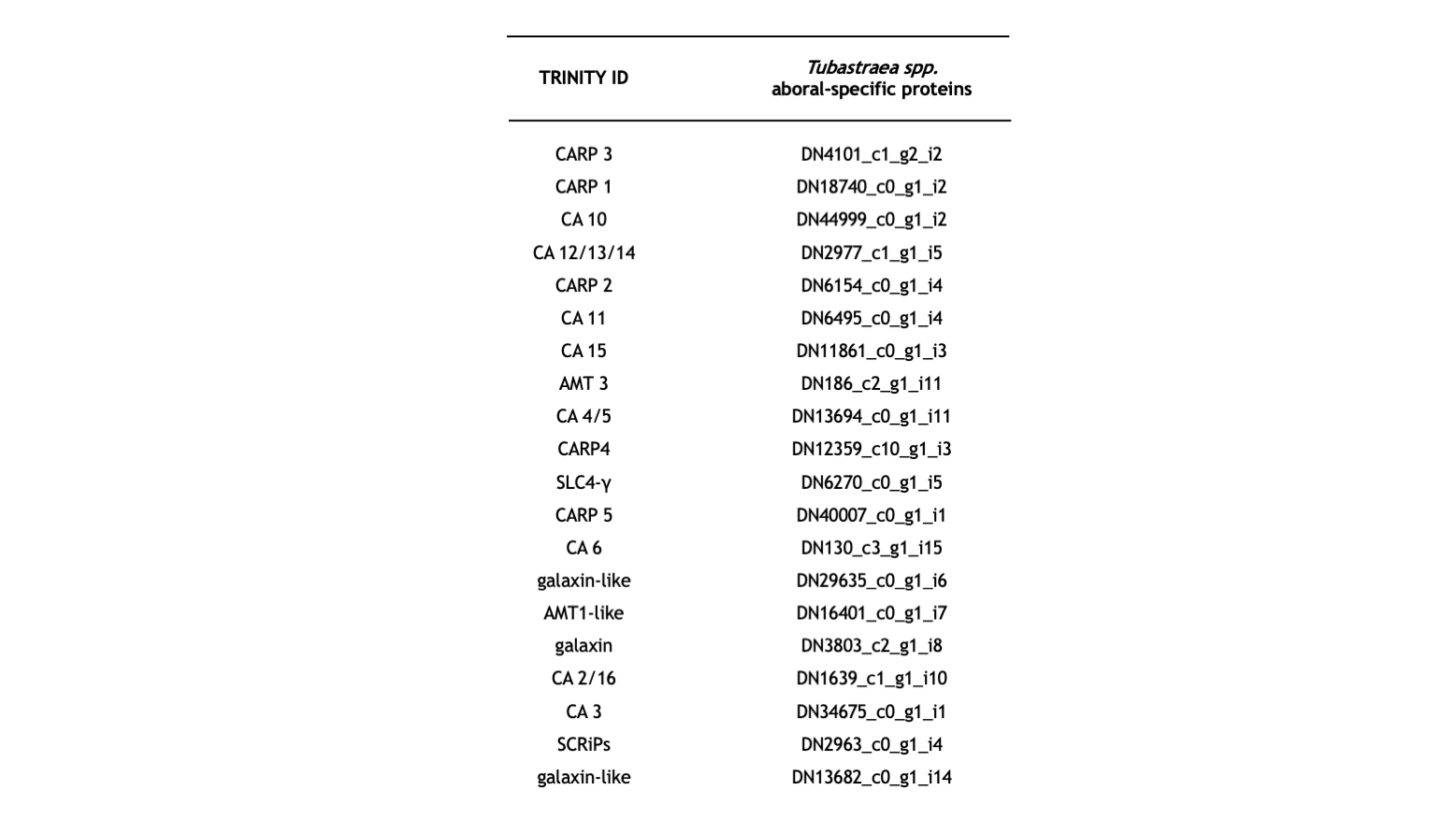

Supplement: Supplementary file 5 — Supplementary Information 5. [file 41598_2022_17022_MOESM5_ESM.tiff]
