# Supplementary material for: Investigating calcification-related candidates in a non-symbiotic scleractinian coral, Tubastraea spp
Source: Sci Rep. 2022 Aug 6;12:13515. doi: 10.1038/s41598-022-17022-4 (PMC9357087; doi:10.1038/s41598-022-17022-4)
Supplement: Supplementary file 3 — Supplementary Information 3. [file 41598_2022_17022_MOESM3_ESM.docx]

**Supplementary information : figure legends**

**Figure S1**. Maximum likelihood (Phyml, LG + I + G) phylogenetic tree of Coral Acid-Rich Proteins (CARPs) from *Tubastraea spp.*, *Stylophora pistillata*, *Acropora digitifera*, *Amplexidiscus fenestrafer*, *Discosoma sp.*, *Nematostella vectensis*, and *Aiptasia pallida*.

**Figure S2**. Maximum likelihood (Phyml, LG + I + G) phylogenetic tree of carbonic anhydrase proteins (CAs) from *Tubastraea spp.* (DN-) and other cnidarian species. Pink circles comprise *Tubastraea spp.* CAs enriched in the total colony versus the oral fraction. Blue circles comprise *Tubastraea spp.* CAs more enriched in the oral fraction compared to the total colony.

**Table S1**. List of Trinity IDs for *Tubastraea spp.* calcification-related candidate homologs.

**Table S2**. Trinity IDs of *Tubastraea spp.* aboral-specific proteins.
